# Teaching an Old Drug New Tricks: Dexamethasone as an In Vivo Inhibitor of Glioblastoma Dispersal

**DOI:** 10.7759/cureus.7749

**Published:** 2020-04-20

**Authors:** Arthur Carminucci, Rut Tejero, Yong Huang, Shabbar Danish, Roland H Friedel, Ramsey Foty

**Affiliations:** 1 Neurosurgery, Rutgers Robert Wood Johnson Medical School, Piscataway, USA; 2 Neuroscience, Mount Sinai School of Medicine, New York, USA; 3 Neuroscience, Icahn School of Medicine at Mount Sinai, New York, USA; 4 Neurosurgery, Mount Sinai School of Medicine, New York, USA; 5 General Surgery, Rutgers Robert Wood Johnson Medical School, New Brunswick, USA

**Keywords:** glioblastoma, dexamethasone, fibronectin matrix assembly, tumor cell dispersal

## Abstract

Identifying drugs that can mitigate dispersal of glioblastoma cells, particularly after patients undergo radiotherapy and concomitant chemotherapy, may increase the length of time to recurrence and improve overall survival. Previous studies have shown that dexamethasone (Dex), a drug currently used to treat brain tumor-related edema, which is tapered immediately after the edema has resolved, induces fibronectin matrix assembly (FNMA) and reduces dispersal of primary human glioblastoma multiforme (GBM) cells in vitro and ex vivo. Here, we utilized an in vivo mouse retina dispersal assay to demonstrate that Dex also inhibits dispersal in vivo. We show that 1) Dex significantly reduces z-axis penetration of glioblastoma cells into mouse retina; 2) treatment alters the morphology of dispersal; 3) without Dex, the presence of fibronectin increases dispersal; 4) treatment activates in vivo FNMA by glioblastoma cells, leading to the containment of the tumor mass; and 5) Dex-mediated activation of FNMA is fibronectin dose-dependent. Dispersal inhibition could be achieved at human equivalent doses as low as 1 mg/day, a dose significantly lower than currently used to reduce edema. This is the first step towards future studies in which patients can be potentially maintained on low-dose dexamethasone therapy with the aim of increasing the time between initial resection and recurrence.

## Introduction

Glioblastoma multiforme (GBM) is a highly aggressive disease with a poor overall prognosis. A clinical hallmark of GBM is its capacity for early and continued dispersal throughout the brain parenchyma [1]. It is this dispersal that, in part, renders this disease resistant to localized therapy [2]. Despite multimodal therapy combining surgical resection, radiotherapy, and adjuvant chemotherapy (Stupp protocol), recurrence is inevitable with a median progression-free survival of approximately eight months [3-4]. Identifying therapies that can mitigate dispersal, particularly after patients undergo the Stupp protocol, may increase the length of time to recurrence and improve overall survival.

The corticosteroid, dexamethasone (Dex), is the standard treatment for vasogenic edema associated with GBM [5]. Following surgery, Dex is typically tapered rapidly to avoid the side effects associated with prolonged administration of high-dose steroids [6]. In addition to its anti-edema properties, there is evidence that Dex has a direct inhibitory effect on GBM growth and proliferation [7]. Previous work in our laboratory indicated that Dex is also capable of inhibiting tumor cell dispersal in vitro and ex vivo [8-9]. The anti-dispersal effects of Dex are exerted through the formation of fibronectin matrix assembly (FNMA), which acts as a “glue” between GBM cells. Dex-associated activation of α5 integrin results in a conformational change in bound fibronectin, resulting in the formation of an insoluble matrix. Dex-induced formation of FNMA in conventional two-dimensional (2D) cultures and three-dimensional (3D) spheroids of human primary GBM cells results in increased strength of the cell-extracellular matrix (ECM) adhesion, increased cell-cell cohesion, and decreased cell motility [8]. Similarly, Dex-mediated inhibition of tumor cell migration was demonstrated ex vivo with GBM neurospheres on human brain slices [9].

To further validate the anti-dispersal effects of Dex on GBM cells, we developed a novel xenotransplantation assay to assess whether Dex treatment could activate FNMA and reduce dispersal in vivo. Interestingly, many commercially available and widely-used human GBM cell lines do not disperse when injected into mouse brains [10]. Additionally, many of the established GBM cell lines are high-passage and have been grown in conventional 2D culture for decades, a condition that has been demonstrated to significantly alter cell physiology and gene expression patterns [11]. Accordingly, such lines may no longer accurately reflect the same biology and, more importantly, the clinical behavior of the initial tumor [12]. Therefore, we sought to develop an in vivo model to study GBM dispersal using low-passage primary human GBM cells. Earlier ex vivo studies in our lab have identified the mouse retina as a potential surrogate substrate to study single-cell GBM dispersal [9]. There are several advantages to using a retina model; the retina is neural tissue and can approximate the physical microenvironment of the GBM cells in the brain [13]. GBM cells spread more readily on the mouse retina, with dispersal beginning as soon as 24 hours post-implantation, compared to ≥ 4 weeks in the mouse brain. The retina is an immune-privileged site, making transplant rejection less likely. Lastly, once extracted, the retina is a flat structure and amenable to optical sectioning using confocal microscopy [14-15].

Accordingly, the aim of this study was to utilize an in vivo retina model of GBM dispersion to study the effect of Dex on tumor cell dispersal. We first compared the capacity for dispersal of two primary GBM lines in the mouse brain and retina transplantation models. We then assessed whether Dex treatment activates FNMA by GBM cells injected into mouse retinas. We explored the association between the fibronectin dose, FNMA and dispersal, and utilized an FNMA-blocking peptide to directly connect matrix assembly to dispersal. Finally, we established the lowest dose of Dex required to activate FNMA and reduce dispersal in our in vivo mouse retina model.

## Materials and methods

Mice

All animal work was performed under the guidance and recommendations in the Guide for the Care and Use of Laboratory Animals of the National Institutes of Health. Mice experiments were approved by the Rutgers-Robert Wood Johnson Medical School (RWJMS) Institutional Animal Care and Use Committee (IACUC) under protocol #16-002 and the Icahn School of Medicine IACUC under protocol #LA11-00401. Male and female C57BL/6J mice (The Jackson Laboratory, Bar Harbor, ME) were used for retina transplant experiments and ICR-SCID mice (IcrTac:ICR-Prkdcscid) (Taconic Biosciences, Hudson, NY) were used for intracranial injections.

GBM cells

Primary human GBM cell lines, GBM 1-4, were generated with approval of the Rutgers-RWJMS Institutional Review Board under protocol # CINJ 001208. Cell lines were prepared and cultured as previously described [8]. These cell lines have previously been published in in vitro and ex vivo experiments [8-9, 16]. GBM-3 has been shown to be the most dispersive in vitro when placed as spheroids on tissue culture plastic, as well as ex vivo on mouse retina and mouse and human brain slices [8-9]. Thus, the GBM-3 cell line was chosen for this in vivo study.

Mouse brain injections

For intracranial transplants, 1 x 10^5^ GBM cells in 2 µl phosphate-buffered saline (PBS) with 2% methylcellulose (Sigma M7027; for increased viscosity) were stereotactically injected into the striatum (Paxinos coordinates: +2 mm lateral, -0.5 mm AP, -3 mm vertical) of adult immunocompromised ICR-SCID mice.

Mouse brain immunohistochemistry

Mice were perfused with PBS and then 4% paraformaldehyde (PFA)/PBS. Brains were fixed overnight in 4% PFA/PBS and then prepared for cryosectioning by two overnight incubations with 12.5% and 25% sucrose/PBS, respectively, and embedding in OCT TissueTek media. Cryosections were cut at 25 µm thickness and stored as floating sections in PBS at 4°C.

For immunofluorescence staining, sections were blocked for one hour (blocking buffer: PBS with 5% donkey serum and 0.3% Triton X-100), then incubated overnight with primary antibodies (Table 1) in antibody dilution buffer (PBS with 1% bovine serum albumin (BSA) and 0.3% Triton™ X-100), followed by staining with AlexaFluor-labeled secondary antibodies (Jackson ImmunoResearch, Westgrove, PA) for two hours, and nuclear counterstaining with 4′, 6-diamidino-2-phenylindole (DAPI) (Invitrogen, Carlsbad, CA). Sections were washed in PBS and mounted with Fluoromount G™ (Southern Biotech, Birmingham, AL). Images were captured with the Zeiss AXIO Imager A2 microscope with AxioCam MRc camera and AxioVision software (Carl Zeiss Meditec AG, Jena, Germany).

**Table 1:** Primary Antibodies for Mouse Brain Section Immunohistochemistry IHC: immunohistochemistry

Antibody (host species)	Clone	Source and order number	Usage
anti-CD31 (rat)	MEC 13.3	BD Biosciences 553370	1:300 for IHC
anti-beta1 Integrin (mouse)	TS2/16	Santa Cruz sc-53711	1:100 for IHC
anti-human nuclear antigen (mouse)	235-1	Millipore MAB1281	1:400 for IHC

Mouse retina injections

GBM-3 cells were stained with PKH-2 green-fluorescent membrane intercalating dye, as previously described [8]. Cell concentration was adjusted to 5x10^4^ cells/ml in normal saline. Human fibronectin was added to the cell suspension at doses ranging from 30 μg/mL to 300 μg/mL. Additionally, 5% methylcellulose was added to increase the overall viscosity of the solution and promote the clustering of cells at the injection site. Where required, 1 mg/ml of functional upstream domain (FUD) fragment or iii-11C peptide were included. FUD (pUR-4) and the iii-11C peptides were obtained from Dr. Jane Sottile through a Uniform Biological Material Transfer Agreement (UBMTA) between the University of Rochester and Rutgers-RWJMS. In preparation for retinal injection, mice were anesthetized with intraperitoneal injections of 100 mg/kg ketamine and 10 mg/kg xylazine. The retinal injection was performed as previously described [17]. In brief, mice were positioned under a dissecting microscope, with eyes facing upward. The skin parallel to the right eyelid was then gently retracted allowing for a slight proptosis of the eye. A 22-gauge beveled needle was then used to make a small puncture through the superior aspect of the cornea just anterior to the limbus. A 25-gauge, 1 μL, blunt tip Hamilton syringe attached to a micromanipulator was then advanced through the puncture site in 1 μm increments. The needle was advanced until resistance was felt, indicating the needle had reached the retina. Next, 2.5x10^4^ cells in 0.5 μL normal saline containing fibronectin and methylcellulose alone, or along with FUD or iii-11C, were injected. Following injection, the needle was left in place for 30 seconds then retracted slowly to avoid scattering of the tumor cells along the injection tract. Bacitracin was applied to the eye to prevent infection. The procedure was then repeated on the left eye. Mice were allowed to recover from the anesthesia and were monitored until awake.

Dexamethasone treatment

Following recovery, dexamethasone or vehicle control treatment was initiated on postoperative day 0. Treatment groups consisted of seven to 12 mice that were injected subcutaneously with doses of 0.015 mg/kg to 0.12 mg/kg of dexamethasone daily for five days. Control groups consisted of mice who were injected subcutaneously with vehicle control consisting of normal saline daily for five days. 

Isolation of mouse retinas

Retinas were isolated as previously described [18]. In brief, following completion of the five-day treatment course, mice were sacrificed by CO_2_ inhalation followed by cervical dislocation. Eyes were removed and fixed in 4% paraformaldehyde under rotation at 4°C for 24 hours. Once fixed, the cornea of the eye was sharply dissected circumferentially, the lens was removed, and the neural retina was gently dissected free from the underlying pigmented retina. Retinas were then mounted on glass slides for immunohistochemistry and microscopy.

Mouse retina immunohistochemistry

FNMA was assessed by immunofluorescence by incubating retinas with primary anti-fibronectin antibody (ab6584) (Abcam, Cambridge, MA) for one hour at room temperature (RT), followed by an Alexa Fluor-568-conjugated secondary antibody (Thermo Fischer Scientific, Waltham, MA) for 30 minutes, with three PBS washes in between. DAPI was added prior to mounting. Images were acquired for this study at the Advanced Microscopy Shared Resource of Rutgers Cancer Institute of New Jersey. Fixed retina tissue was imaged using a Nikon A1R-Si Confocal Microscope System (Nikon Instruments Inc., Melville, NY). Image analysis was performed using the Nikon Elements version 4.30 acquisition software. Z-axis dispersal of GBM cells into mouse retinas was determined by acquiring 1 μm optical sections through the retina. Dispersal distance was measured between the superior-most and inferior-most sections containing fluorescent signal. 

Statistical analysis

For comparison of z-axis dispersal between Dex treatment and control, a one-tailed Mann-Whitney U-test was used. Kruskal-Wallis and Dunn’s multiple comparisons tests were used to compare three or more groups. Assumption of normality was not made since not all data sets were found to be normally distributed when assessed by the D’Agostino-Pearson omnibus normality test or by analysis with Q-Q plots. An a of 0.05 was utilized for all statistical tests. Post-hoc power analysis was used to determine whether the sample sizes used, differences between means, and scatter around those means achieved at least 80% power at an α of 0.05.

## Results

A mouse retina model of GBM dispersal 

**Figure 1:**
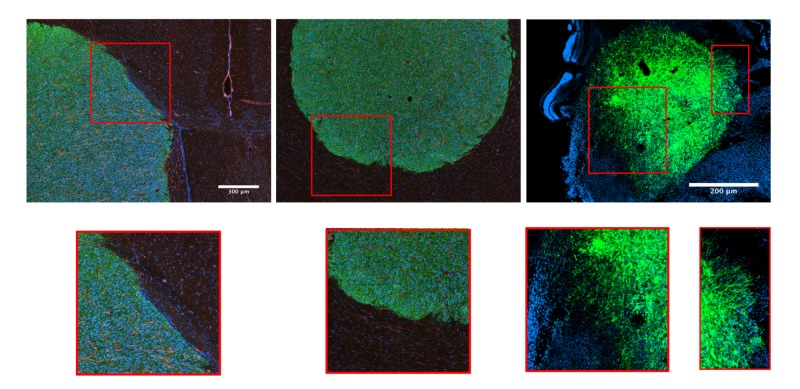
A mouse retina model of GBM dispersal Human primary GBM cell lines were injected either intra-cranially into mouse brains (A, B) or into mouse retinas (C). For intracranial injections, brains were harvested after one month. Immunohistochemistry was then used to detect human GBM cells (b1-integrin, green), endothelial cells (CD31, red), or nuclei (DAPI, blue). For retina injections, GBM cells were stained with PKH2 green fluorescent cell linker prior to injection. After five days, retinas were harvested and counterstained with DAPI. Brain slices and retinas were imaged by fluorescence microscopy. Note the complete absence of GBM cell dispersal in brain tissue (A, B, and insets) and marked dispersal in retina (C and insets). DAPI: 4′, 6-diamidino-2-phenylindole; GBM: glioblastoma multiforme

We have previously shown that freshly-isolated, low-passage, human primary GBM cells readily disperse in vitro and ex vivo and now wished to determine whether they also had the capacity for in vivo dispersal in a mouse brain model. GBM cells were injected into mouse brains. After four weeks, brains were dissected, tissue sections prepared for immunohistochemistry using markers specific for detection of human cells within a mouse neural background. As is evident in Figure 1A-B, GBM cells, labeled for human-specific b1-integrin (green), can grow significantly to form large tumors but do not disperse. Rather, tumors form a sharp boundary with the normal parenchyma. However, when GBM cells are injected into mouse retinas, they appear to readily disperse (Figure 1C), both as single cells (panel C, left inset) and as chords of cells (panel C, right inset), within five days of injection. Accordingly, GBM cells appear to behave in a more clinically relevant manner in the retina model.

Dex-treatment activates FNMA in GBM cells and alters the pattern of retina dispersal

**Figure 2:**
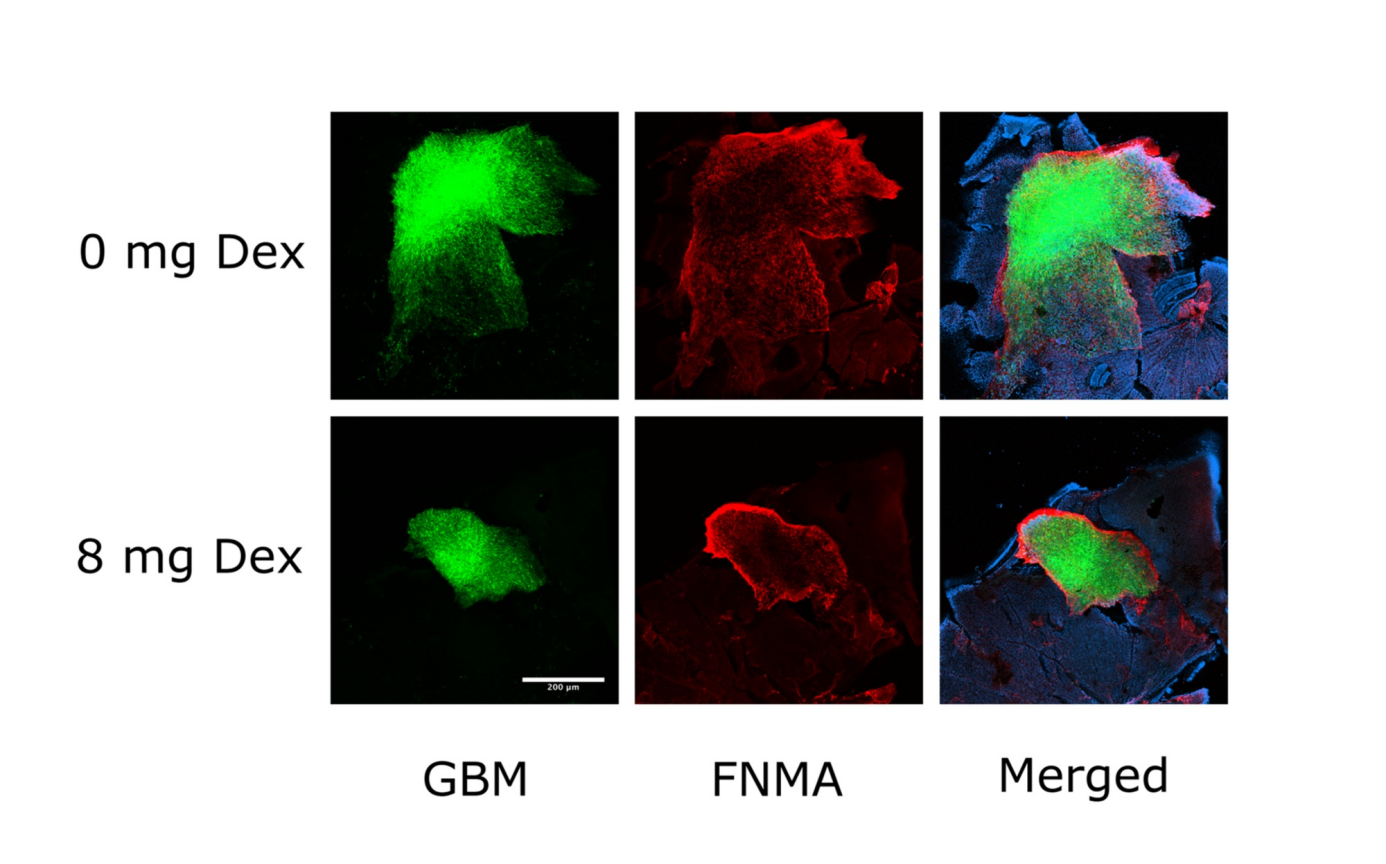
Dex-treatment activates FNMA in GBM cells and alters the pattern of retina dispersal Dex-treatment activates FNMA in GBM cells and alters the pattern of retina dispersal. Dex: dexamethasone; FNMA: fibronectin matrix assembly; GBM: glioblastoma multiforme

Dex has been previously demonstrated to exert a significant inhibitory effect on the dispersal of GBM cells in vitro and ex vivo by activating FNMA, effectively increasing the strength of cell-cell cohesion and cell-ECM adhesion [12-13]. Here, we show in our in vivo mouse retina model that injection of 0.12 mg/kg Dex, a dose that is equivalent to an adult human clinical dose of 8 mg/day, was able to activate FNMA and significantly reduce dispersal of GBM cells (Figure 2). Retinas from untreated mice showed extensive dispersal of GBM cells (green). Fibronectin (red) was detected in untreated retinas; however, relatively little was assembled and was distributed in a pericellular pattern. In contrast, Dex treatment significantly inhibited the dispersal of GBM cells and resulted in the assembly of a fibronectin barrier surrounding the injected GBM cells.

Dex activity requires the presence of soluble fibronectin

**Figure 3:**
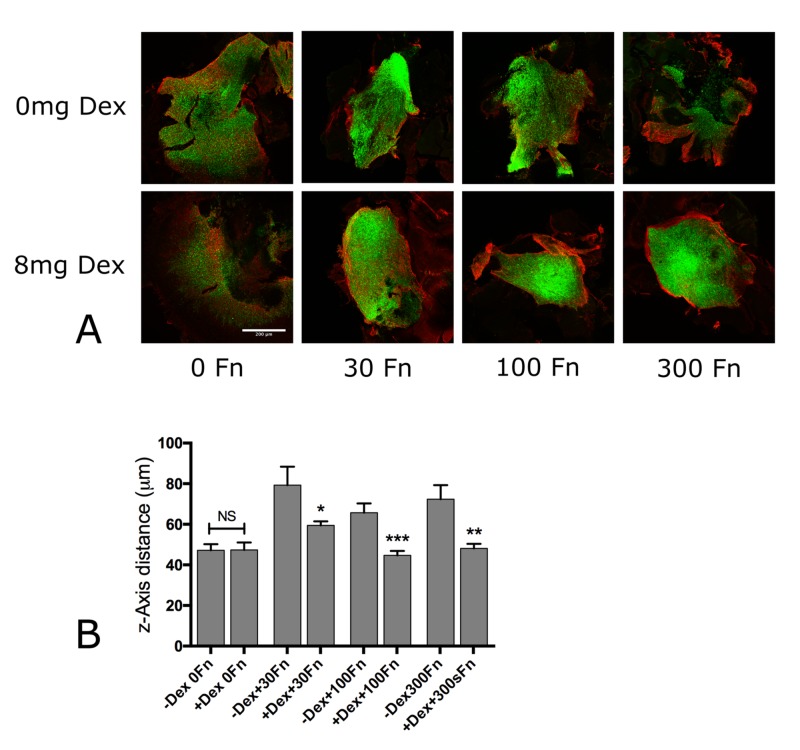
Dex activity requires the presence of soluble fibronectin (A) GBM cells were co-injected either with vehicle control or in the presence of 30 - 300 mg/ml soluble human fibronectin and mice were treated for five days with HCE, 8 mg Dex/day. GBM cells (green) and FNMA (red) were imaged by epifluorescence microscopy. Thirty mg/ml of fibronectin was sufficient to activate FNMA; however, 300 mg/ml resulted in a more well-developed fibronectin barrier. Scale bar = 200 micrometers. (B) Z-axis dispersal distance of GBM cells into mouse retinas also required the presence of soluble human fibronectin. Mean z-axis dispersal migration distance was compared for the control group and for each fibronectin concentration by the Mann-Whitney U-test. Bars are standard errors of the mean. Asterisks represent the significant differences of p < 0.001 (***), p < 0.01 (**), and p < 0.05 (*). NS denotes p > 0.05. Note the significant reduction of z-axis migration distance in Dex-treated animals but only when the fibronectin was present. Dex: dexamethasone; FN: fibronectin; FNMA: fibronectin matrix assembly; GBM: glioblastoma multiforme; HCE: human clinical equivalent; NS: not significant

In the absence of Dex, fluorescently-labeled GBM cells, when injected into mouse retinas without supplemental fibronectin, do not assemble a fibronectin matrix. With the injection of 0.12 mg/kg of Dex, however, fibronectin concentrations as low as 30 mg/ml appeared to activate matrix assembly. This process becomes more robust as the fibronectin concentration increases from 30 - 300 mg/ml with the formation of a fibronectin barrier surrounding the injected GBM cells (Figure 3A). Dispersal was quantified by measuring the z-axis migration distance of GBM cells in confocal image stacks of retinas of untreated and Dex-treated mice. Mean z-axis migration distance was compared by a one-tailed, Mann-Whitney U test. In the absence of injected supplemental fibronectin, z-axis migration was statistically similar for untreated and Dex-treated mice (p = 0.3896). In contrast, co-injection of 30 mg/ml of soluble fibronectin significantly reduced dispersal in Dex-treated mice (p = 0.0307). Co-injection of 100 mg/ml of fibronectin further reduced dispersal in the retinas of Dex-treated animals (p = 0.0008 and p = 0.0014), respectively (Figure 3B).

Blocking Dex-mediated activation of FNMA by the FUD fragment of fibronectin reinstates dispersal of GBM cells in vitro and in vivo 

**Figure 4:**
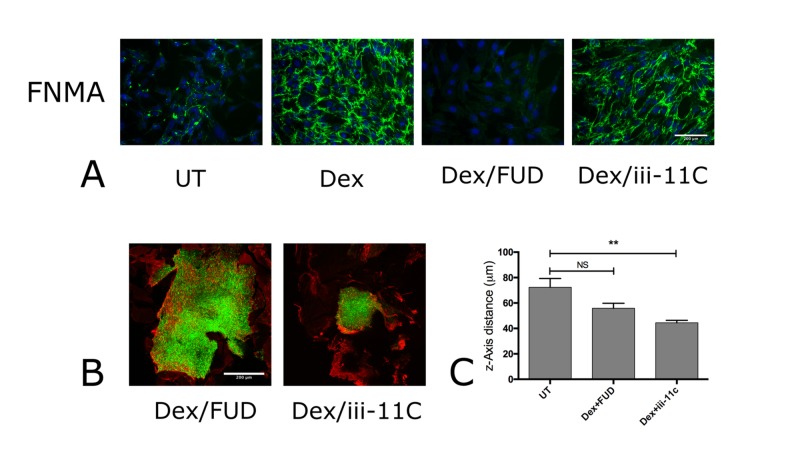
Blocking Dex-mediated activation of FNMA by the FUD fragment of fibronectin promotes dispersal of GBM cells in vitro and in vivo (A) GBM cells in conventional 2D culture were treated with Dex and incubated either in the presence of the FUD fragment of fibronectin or in its control peptide (iii-11C). After 24 hours in culture, FNMA was assessed by immunohistochemistry. Note the absence of FNMA by the FUD fragment relative to the iii-11C peptide. The effect of FUD and iii-11C on actin organization was also assessed. In contrast to its effect on FNMA, FUD did not effectively alter Dex-induced actin stress fiber formation, as has previously been reported by Shannon et al. [8]. (B) Co-injection of FUD, but not iii-11C promotes in vivo GBM dispersal. Mouse retinas were injected with fluorescently-labeled GBM cells (green) either in the presence of FUD or control peptide, iii-11C. Mice were treated with Dex for five days, whereupon retinas were extirpated and stained for FNMA (red). Note the absence of FNMA and significant dispersal of GBM cells when FUD was co-injected. In contrast, the co-injection of iii-11C appears to have no effect on restoring dispersal. Note also the presence of the FNMA barrier. Scale bar = 200 micrometers. (C) Mean z-axis dispersal migration distance was compared for retinas co-injected with the vehicle, FUD-fragment, or iii-11C by the Kruskal-Wallis and Dunn’s multiple comparisons test. Comparisons were between untreated (UT) and Dex+FUD or UT and Dex+iii-11C.  Bars are standard errors of the mean. Asterisks represent significant difference of p < 0.01 (**), and p < 0.05 (*). NS denotes p > 0.05. Note that only co-injection of the FUD fragment significantly restores GBM dispersal to levels approaching those of untreated animals. Dex: dexamethasone; FNMA: fibronectin matrix assembly; FUD: functional upstream domain; NS: not significant; 2D: two-dimensional

To directly connect the Dex-mediated assembly of fibronectin matrix to reduced dispersal (in vivo), we co-injected mice retinas with GBM cells, 300 mg/ml fibronectin, and either the matrix-blocking FUD fragment or control peptide iii-11C. To determine effective dose, we first performed FNMA blocking assays in conventional 2D culture. Complete elimination of Dex-mediated activation of fibronectin matrix assembly was achieved by incubation of GBM cells with 1 mg/ml of FUD. A similar concentration of the iii-11C control peptide had no effect on matrix assembly (Figure 4A). We then asked whether co-injecting fibronectin and 1 mg/ml FUD or iii-11C into mouse retinas in Dex-treated animals would eliminate the matrix assembly and reduce dispersal in vivo. FUD-treated retinas demonstrated extensive dispersal of GBM cells and little to no matrix assembly, whereas retinas co-injected with the control peptide, iii-11C, gave rise to a compact GBM cluster surrounded by a fibronectin barrier (Figure 4B). Moreover, the mean z-axis migration distance of GBM cells was found to be significantly different between untreated (UT) animals and those co-injected with iii-11C and treated with Dex (Kruskal-Wallis test, p = 0.0036, and Dunn’s Multiple Comparisons test, p = 0.01), but not with Dex-treated animals co-injected with the FUD fragment (Figure 4C). These data demonstrate a direct connection between the fibronectin matrix assembly and GBM dispersal in vivo.

In vivo inhibition of GBM dispersal by low-dose Dex-treatment

**Figure 5:**
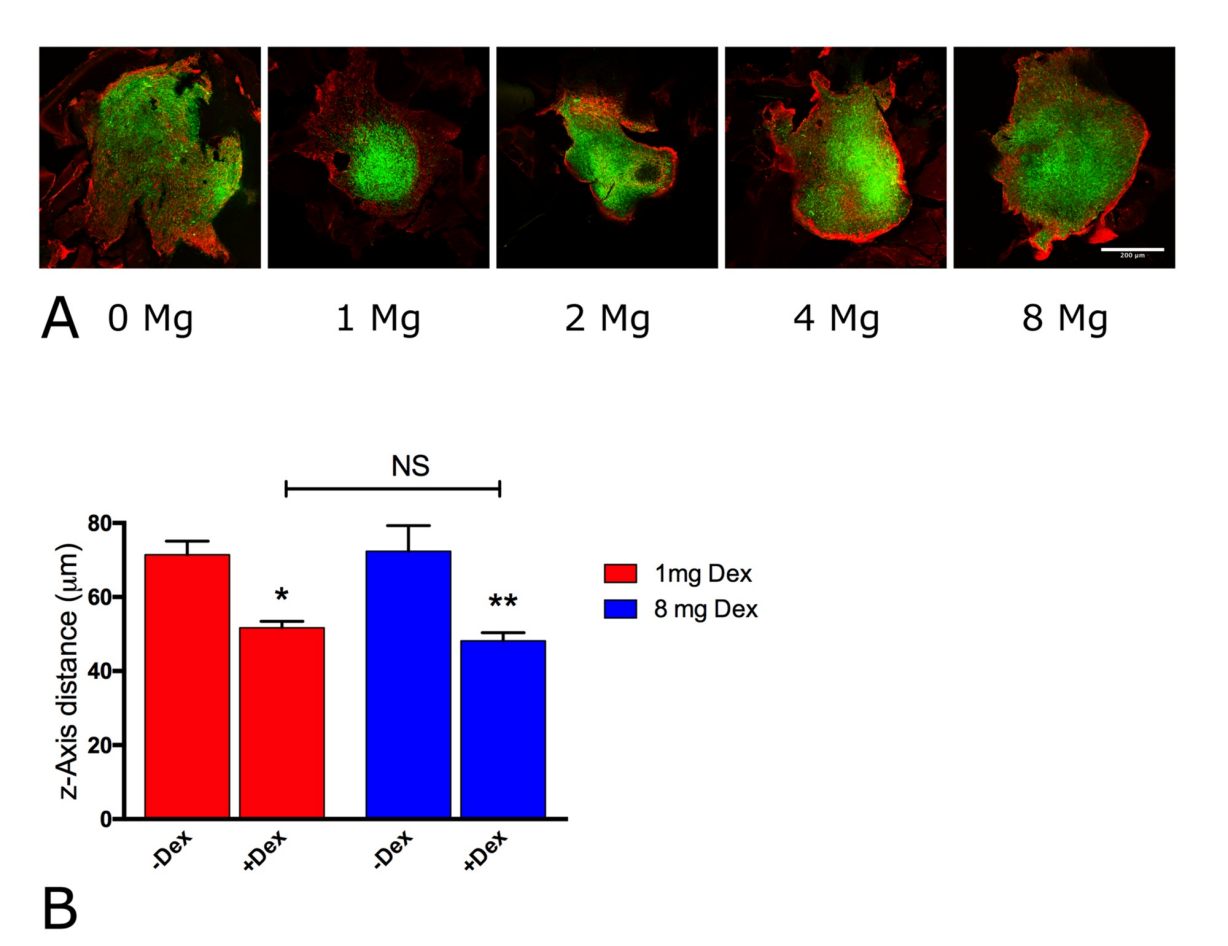
In vivo inhibition of GBM dispersal by low-dose Dex-treatment (A) Fluorescently-labeled GBM cells (green) were co-injected with 300 ug/ml of fibronectin into mouse retinas. Mice were treated with either HCE 1, 2, 4, or 8 mg/day for five days, whereupon retinas were extirpated and stained for FNMA (red). Dex doses as low as 2 mg/day were sufficient to activate FNMA and reduce dispersal. (B) Z-axis migration distance between 1 mg/day and 8 mg/day Dex treatment by Kruskal-Wallis and Dunn’s multiple comparisons tests. Bars are standard errors of the mean. Asterisks represent significant difference of p < 0.01 (**) and p < 0.05 (*). NS denotes p > 0.05. Whereas both 1 mg/day and 8 mg/day Dex treatments reduced z-axis migration, there was no significant difference in migration between the two doses. Dex: dexamethasone; FNMA: fibronectin matrix assembly; GBM: glioblastoma multiforme; HCE: human clinical equivalent; NS: not significant

In GBM, Dex is typically used at relatively high doses to control brain tumor-related edema [5]. We wished to determine the lowest possible in vivo dose that could activate the fibronectin matrix assembly. GBM cells were injected into mouse retinas and mice were subsequently treated with vehicle control or 0.015, 0.03, 0.06, or 0.12 mg/kg Dex doses, equivalent to 1, 2, 4, and 8 mg/day human adult doses, for five days, whereupon retinas were extirpated, stained for FNMA, and imaged by confocal microscopy. As can be seen in Figure 5A, a dose equivalent to 8 mg/day Dex resulted in the activation of FNMA and the formation of a fibronectin barrier surrounding the GBM cells. Similarly, a Dex dose as low as an equivalent of 2 mg/day in humans was sufficient to activate FNMA, also resulting in the assembly of a fibronectin barrier. Interestingly, doses as low as 1 mg/day were sufficient to significantly reduce the z-axis migration distance (Kruskal-Wallis, p = 0.00007, Dunn’s MCT = 0.01) without a marked activation of FNMA. A human equivalent dose of 8 mg/day was slightly more effective in reducing z-axis migration distance (Dunn’s multiple comparison test (MCT), p = 0.001). No significant difference was found in the mean z-axis migration distance between animals treated with human equivalent doses of 1 or 8 mg/day of Dex (Figure 5B). This suggests that low-dose Dex may be effective in reducing GBM dispersal at doses significantly lower than those currently used to treat brain tumor-related edema.

## Discussion

Glioblastoma almost always recurs, with a progression-free survival of only seven months [4]. How quickly this happens depends on several factors, including a propensity for dispersal and continued growth. Preventing or impeding tumor cell dispersal or reducing tumor growth rate could delay the onset of recurrence.

Various factors can influence dispersal and growth, including the strength of cell-cell cohesion, cell-ECM adhesion, cell motility, and of the ECM [19-22]. A decrease in the ability of cells to detach from a primary mass (mediated by increased cell-cell cohesion), coupled with an effective decrease in cell motility (mediated by increased cell-ECM attachment), could, therefore, reduce dispersal.

Previous studies from our group have shown that dexamethasone (Dex), a drug routinely used to treat brain tumor-related edema, was highly effective in activating FNMA in human fibrosarcoma (HT-1080), rat prostate cancer cells, and various commonly-used GBM cell lines [19, 23-24]. More recent studies have demonstrated a similar effect for primary GBM cell lines in vitro and ex vivo [8-9]. The current study explores whether Dex can reduce dispersal in vivo.

We used a novel mouse retina model to assess dispersal. This was mainly due to the fact that, when injected into mouse brains, many GBM cell lines either failed to grow or to disperse [10]. This proved to be the case for our primary, low-passage GBM lines. Indeed, two of the lines (GBM-1 and GBM-2) grew but did not disperse, whereas GBM-3 and GBM-4 failed to grow appreciably or disperse in the mouse central nervous system (CNS). These results are inconsistent with the clinical manifestation of GBM in the human brain. Lack of CNS dispersal by all four GBM lines may be due to their propagation in the serum-containing medium. Le et al. have demonstrated that cell lines derived from patient samples, when grown in serum-free neural stem cell medium, can disperse in the brain [25]. Perhaps differences in differentiation states significantly influence CNS dispersal of isolated primary GBM cells. In this study, we asked whether the mouse retina could be used as a surrogate tissue for dispersal assays. We, therefore, injected GBM-3, previously demonstrated to be the most dispersive line in vitro and ex vivo, into mouse retinas and showed that they readily disperse within 24 hours of injection. Accordingly, the retina appears to be a better model for testing dispersal of GBM than the more conventional CNS injection and may be useful for commercial cell lines that typically do not disperse in typical CNS injection studies.

We demonstrated that Dex injected in mice at doses equivalent to those used clinically to control brain tumor-related edema activates FNMA in GBM cells, resulting in the presence of a fibronectin barrier that contained the injected GBM cluster and significantly reduced the z-axis migration of tumor cells (Figure 3). This was also demonstrated in vitro and ex vivo [8-9]. In those studies, FNMA blocked migration by increasing the strength of cell-cell cohesion to levels that impeded detachment of cells from GBM spheroids. In vivo, it is likely that FNMA acted in a similar manner, impeding cells from escaping from the injected cell clump and penetrating the retina tissue in the z-axis. In contrast to the in vitro and ex vivo studies, we were not able to measure lateral migration since it was impossible to normalize the data by the number of injected cells that remained within the injection site. Interestingly, when GBM cells were co-injected with fibronectin into retinas, in the absence of Dex treatment, the fibronectin barrier was not assembled and GBM cells were able to spread both laterally and in the z-axis. Conversely, the addition of Dex resulted in the assembly of the fibronectin barrier and containment of the injected GBM cells. We demonstrated that assembly of the fibronectin matrix was required since blocking Dex-mediated FNMA by co-injecting the FUD fragment effectively restored dispersal. This suggests that in vivo, Dex must, therefore, function, at least in part, by activating α5 integrin activity in deficient GBM cells.

Penetrating brain injury, including tumor resection, triggers the upregulation of fibronectin at the injury site [26]. Potential sources of fibronectin in CNS injuries include reactive astroglia and ingressing fibroblasts or Schwann cells, but plasma fibronectin is also an important source. Interestingly, penetrating brain injury, including tumor resection, triggers the upregulation of fibronectin at the injury site [26]. Increased fibronectin concentrations, in the presence of Dex-activated integrins, could, in principle, provide a microenvironment favorable to increased intercellular cohesion and decreased capacity for dispersal. Our study suggests that a strategy in which Dex is administered immediately after resection and continued thereafter could reduce the dispersal of recurrent tumors, possibly keeping them contained and more amenable to localized therapy.

Dexamethasone is considered the “gold standard” for the treatment of tumor-associated cerebral edema. Despite its widespread use, the exact therapeutic mechanisms of Dex are not fully understood. It is suggested that the anti-edema effect of dexamethasone likely results from the reduction of blood vessel permeability and restoration of the blood-brain-barrier [7]. The anti-proliferative and anti-dispersal effects demonstrated by Dex are possibly induced from genomic changes within the GBM cells by activation of the glucocorticoid receptor [27]. Dex is frequently administered in divided doses totaling 4 - 24 mg daily [28]. Following surgery, standard practice is to taper Dex over a one to a four-week period to avoid potential adverse side-effects, such as cushingoid appearance, immunosuppression, glucose intolerance, hypertension, and osteoporosis. The severity of side effects is often dependent on the duration of use and dose of the steroid given [27]. Typically, Dex is only restarted at a low dose (0.5 - 1 mg daily) if patients become symptomatic or have a poor life expectancy secondary to recurrence [6]. In some cases, Dex may be given during the temozolomide/radiation treatment period to relieve treatment-related side effects, such as nausea and vomiting.

Recent studies indicate that Dex-induced anti-proliferative effects may, in fact, protect GBM cells against radiotherapy and chemotherapy when given concomitantly [29], supporting the concept that it should be tapered off well before therapy begins. Moreover, Luedi et al. showed that the drug increases invasion, proliferation, and angiogenesis in human GBM stem cell-derived orthotopic tumors, potentially worsening GBM patients’ prognoses [30]. However, in the latter study, the authors used 50 μM Dex in their in vitro studies and 2 mg/kg Dex in their in vivo mouse studies. These doses are 500 times higher than the dose used in our previous in vitro and ex vivo assays [8-9], and approximately 17 - 67 times higher than the in vivo doses used in this study. For this reason, it is not possible to directly compare the dispersal inhibitory effect of low-dose Dex demonstrated here with the apparent invasion-stimulatory effect observed in Luedi et al.

**Figure 6:**
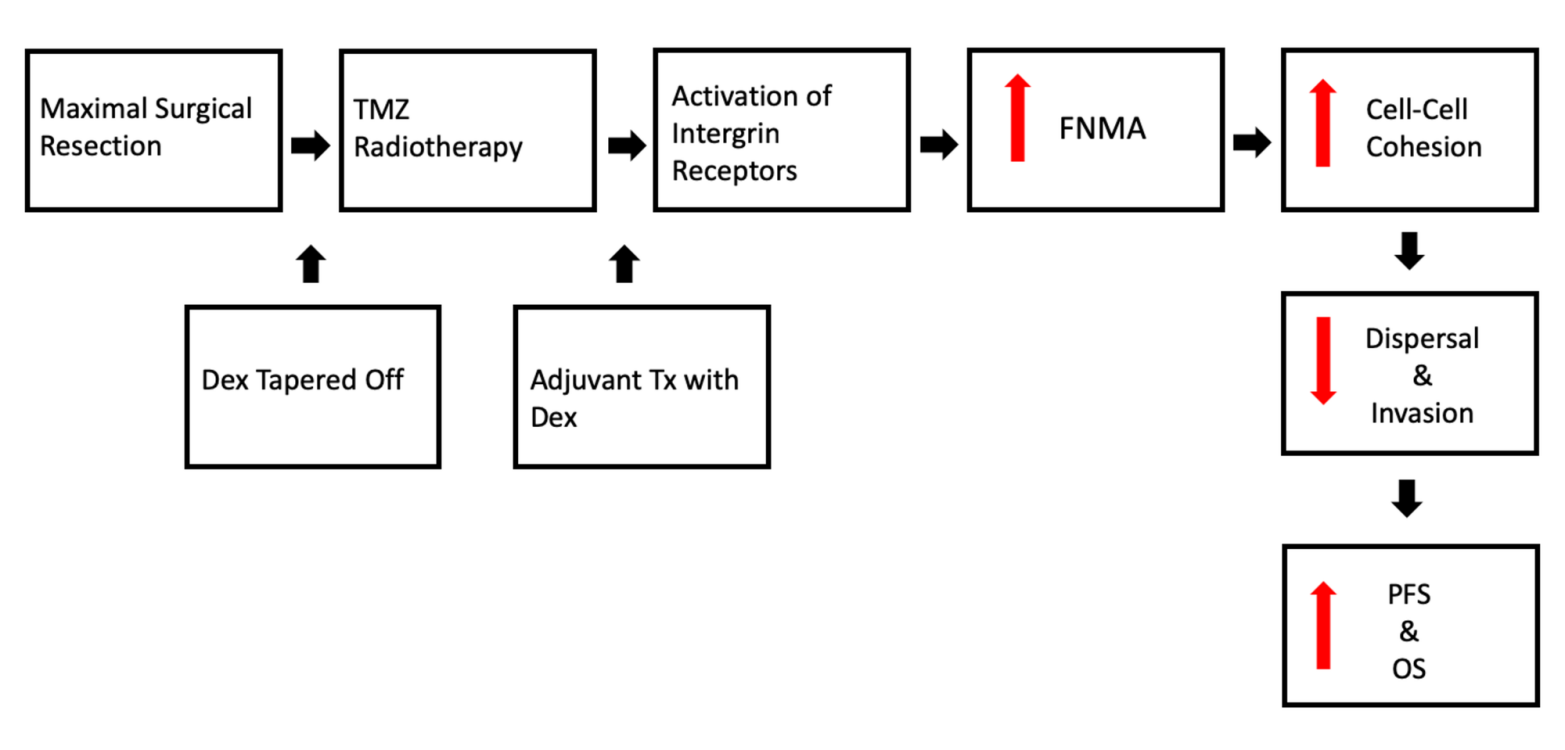
Theoretical treatment paradigm Dex: dexamethasone; FNMA: fibronectin matrix assembly; OS: overall survival; PFS: progression-free survival; TMZ: temozolomide; Tx: treatment

Few treatment options exist for patients once they complete their standard treatment with the Stupp protocol. We propose a possible translational paradigm in which patients with GBM receive their standard surgical resection and adjunctive temozolomide (TMZ) and radiation, followed by the initiation of adjuvant low-dose dexamethasone (Figure 6). We hypothesize that continued low-dose Dex will activate integrin receptors, resulting in the generation of a fibronectin matrix, leading to increased intercellular cohesion, decreased dispersal, and ultimately, increasing progression-free survival. We recognize the potential of adverse side effects from the prolonged administration of corticosteroids. However, with close monitoring and consultation with an endocrine specialist, if necessary, any potential side effects can be successfully managed. Further pre-clinical studies are needed to determine if the Dex-integrin-FNMA relationship can increase overall survival in an animal brain model. Nevertheless, targeting GBM tumor cell dispersal through the extracellular matrix warrants further investigation.

## Conclusions

Our study defines a role for fibronectin as a facilitator of primary glioblastoma multiforme (pGBM) dispersal and Dex-mediated activation of FNMA as an inhibitor of that process. We have shown that Dex significantly reduces z-axis penetration of pGBM cells into the mouse retina, Dex treatment significantly alters the morphology of dispersal of injected pGBM cells within the x,y plane, without Dex, the presence of fibronectin increases dispersal, Dex treatment activates FNMA by pGBM cells leading to the containment of the tumor mass, and Dex-mediated activation of FNMA is fibronectin dose-dependent. Moreover, treatment with FUD (but not iii-11C) restores the ability of tumor cells to disperse. Interestingly, dispersal inhibition could be achieved at Dex doses as low as 1 mg/day. 
